# Ferroptosis is involved in regulating perioperative neurocognitive disorders: emerging perspectives

**DOI:** 10.1186/s12974-022-02570-3

**Published:** 2022-09-06

**Authors:** Yanhong Song, Ziyi Wu, Hang Xue, Ping Zhao

**Affiliations:** Department of Anesthesiology, Shengjing Hospital of China Medical University, Shenyang, 110004 China

**Keywords:** Ferroptosis, Perioperative neurocognitive disorders, Neurotoxicity

## Abstract

Since the twenty-first century, the development of technological advances in anesthesia and surgery has brought benefits to human health. However, the adverse neurological effects of perioperative-related factors (e.g., surgical trauma, anesthesia, etc.) as stressors cannot be ignored as well. The nervous system appears to be more “fragile” and vulnerable to damage in developing and aging individuals. Ferroptosis is a novel form of programmed cell death proposed in 2012. In recent years, the regulation of ferroptosis to treat cancer, immune system disorders, and neurodegenerative diseases have seen an unprecedented surge of interest. The association of ferroptosis with perioperative neurocognitive disorders has also received much attention. Cognitive impairment can not only affect the individual’s quality of life, but also impose a burden on the family and society. Therefore, the search for effective preventive and therapeutic methods to alleviate cognitive impairment caused by perioperative-related factors is a challenge that needs to be urgently addressed. In our review, we first briefly describe the connection between iron accumulation in neurons and impairment of brain function during development and aging. It is followed by a review of the pathways of ferroptosis, mainly including iron metabolism, amino acid metabolism, and lipid metabolism pathway. Furthermore, we analyze the connection between ferroptosis and perioperative-related factors. The surgery itself, general anesthetic drugs, and many other relevant factors in the perioperative period may affect neuronal iron homeostasis. Finally, we summarize the experimental evidence for ameliorating developmental and degenerative neurotoxicity by modulating ferroptosis. The suppression of ferroptosis seems to provide the possibility to prevent and improve perioperative neurocognitive impairment.

## Introduction

With health issues intensifying around the world, worldwide surgical procedures are increasing dramatically and surgery has become one of the predominant means of curing disease. Global surgical volume statistics published by the World Health Organization (WHO) have shown that the global surgical volume is up to 320 million each year and is gradually growing [1]. With the dual effect of improved surgical techniques and safety, surgical treatment has made contributions to human health. However, brain dysfunction caused by perioperative-related factors should not be neglected equally. Perioperative neurocognitive disorders (PND) are a common postoperative complication of the central nervous system, mainly including postoperative cognitive dysfunction (POCD) and postoperative delirium. It is usually characterized by confusion, anxiety, personality changes, and memory impairment [2]. Currently, the pathogenesis of PND is unclear and may be the result of a combination of factors. Oxidative stress, apoptosis, neuroinflammation, changes in synaptic properties, and abnormal accumulation of amyloid β (Aβ) may all be involved in the pathological development of PND [3, 4]. Compared to other age groups, elderly patients have a lower resistance to stress and a relatively lower tolerance to surgery, resulting in a higher incidence of PND [5]. It is important to note that PND is not a harmless disease. Not only does it lead to longer hospital length of stay, higher costs, and increased readmission rates, but it may also increase 5-year mortality after surgery [6, 7]. In addition, postoperative delirium may accelerate the deterioration of cognitive function in elderly patients and is associated with a decline in neurocognitive function in the distant postoperative period [8, 9]. Cognitive impairment not only leads to a reduced level of quality of life for the individual, but may also place a heavy burden on the family and society. Aging-induced neurological degeneration increases susceptibility to perioperative cognitive impairment, and the developing brain is likewise more vulnerable to multiple factors such as anesthetics and surgical trauma. Experimental animal studies have found that some anesthetics (e.g., isoflurane, sevoflurane, ketamine, sodium thiopental, and others) are neurotoxic to the developing brain [10, 11], and general anesthesia in infancy may also be associated with impaired recall memory in childhood [12]. Therefore, effective prevention and mitigation of anesthetic developmental neurotoxicity and perioperative neurocognitive impairment have become urgent issues to be addressed.

Ferroptosis is a novel iron-dependent form of programmed cell death proposed in 2012 [13], which is characterized by the generation of lipid peroxides by highly expressed unsaturated fatty acids in the cell membrane and the accumulation of lipid peroxides to lethal levels catalyzed by divalent iron or ester oxygenases, thereby inducing cell death [14, 15]. Iron is an essential trace element in the human body, involved in regulating energy metabolism, transporting oxygen, DNA synthesis, and improving immunity. In nerve cells, iron is not only engaged in mitochondrial respiration, but also plays a significant role in the synthesis of neurotransmitters and myelin sheaths, which is an indispensable part of maintaining normal physiological functions of the brain [16]. Elevated iron levels in the brain can damage nerve cells by inducing lipid peroxidation, mitochondrial inactivation, and neuroinflammation. Current research has demonstrated that iron overload can be linked to a variety of neurodegenerative diseases such as Alzheimer's disease, Parkinson’s disease, and epilepsy [17, 18]. And the mechanisms of general anesthetic neurotoxicity have similarities with neurodegenerative diseases. Ferroptosis, a form of cell death, may inevitably disrupt nerve cell function. However, this is not the only cause of the cognitive decline. Inhibition of neuronal activity can also affect cellular function. The intensity of perioperative stimuli and the susceptibility of neurons may lead to different outcomes for individual perioperative cognitive decline. Some individuals can return to normal quickly, while others take 6 months to 1 year, or even longer. Emerging insights suggest that ferroptosis is possibly involved in the pathogenesis of PND. Our review provides an update on the targeted modulation of ferroptosis to attenuate cognitive impairment attributed to perioperative-related factors.

### Mechanisms of ferroptosis

**Fig. 1:**
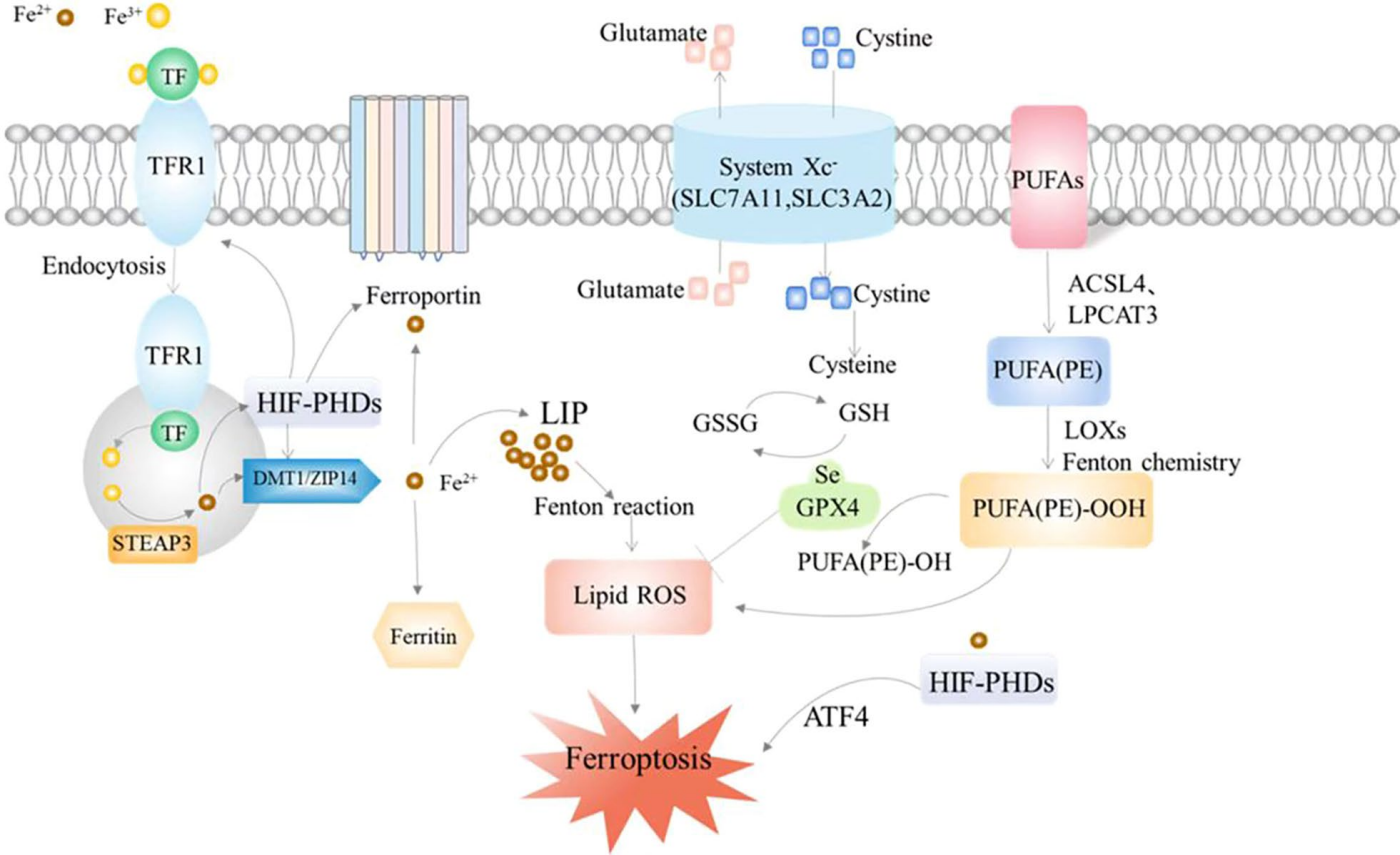
The main mechanisms regulating ferroptosis

Ferroptosis is a novel form of cell death that differs from apoptosis, autophagy, and necrosis, as proposed by Dixon et al. in 2012 [13]. It can be evoked by endogenous and exogenous pathways, with the endogenous pathway generally referring to the suppression of intracellular antioxidant enzyme activation, while the exogenous pathway is through the regulation of membrane transport proteins, mainly including the inhibition of System Xc^−^ and activation of transferrin [19]. Increased iron accumulation and lipid peroxidation are critical signals for the induction of ferroptosis, and disruption of the balance between cellular regulation of oxidative damage and antioxidant defense will trigger cellular death. Currently, the precise mechanism of ferroptosis is still unclear and potentially closely interrelated with a variety of biological pathways, the main ones widely recognized being iron, lipid, and amino acid metabolic pathways [20] (Fig. 1).

### Iron metabolism

Iron is an essential micro-element with two oxidation states, Fe^2+^ and Fe^3+^. Fe^3+^ binds to transferrin (TF) and eventually flows into the cells via the transferrin cycle. The principal processes include the binding of Tf–Fe^3+^ to transferrin high-affinity receptor 1 (TfR1) on the cell membrane and its entry into the cell to form an endosome by endocytosis. The acidic nuclear endosomal environment separates Fe^3+^ from TF, while the metal reductase STEAP3 reduces Fe^3+^ to Fe^2+^ [21]. Fe^2+^ can be subsequently assisted by the divalent metal transporter 1(DMT1) or the zinc transporter protein ZIP14 (ZRT/IRT-like protein14) to flow to the cytoplasmic labile iron pool(LIP) [22]. TF and TfR1, which have released Fe^3+^, can be reintroduced to the cell surface by cytosolic action. In this process, Tf and TfR1 can be recycled and reused in the next cycle of cellular iron uptake. Ferroportin 1(FPN1) is the unique protein in humans that transports intracellular iron efflux. Intracellular Fe^2+^ can be oxidized to Fe^3+^ by hephaestin and bound to ferroportin 1, which in turn are transferred out of the cell. Hepcidin can regulate FPN1 levels by binding to FPN1 and triggering its internalization, ubiquitination, and degradation. Iron response element (IRE) is a highly conserved RNA stem–loop structure present at the 5′ end or 3′ end of the non-coding region of iron metabolism-related protein genes (e.g., TFR1, DMT1, ferritin, FPN1, etc.) [23]. Iron regulatory proteins 1 and 2 (IRP1 and IRP2) can act as sensors to control intracellular iron metabolic homeostasis, recognizing and binding IRE in a structure and sequence-specific manner, and thereby regulating iron uptake, storage, and export [24]. When intracellular iron is at relatively low levels, IRP/IRE can increase iron uptake and inhibit iron export by upregulating the expression of TFR1 and DMT1 and downregulating FPN and ferritin [25].

Ferritinophagy is a type of selective cellular autophagy. Nuclear receptor co-activator 4 (NCOA4) has been identified as a crucial regulator of ferritinophagy, which binds to ferritin through the C-terminal helix domain and is delivered to the lysosome for degradation and release of free iron [26]. The regulation of NCOA4 expression by iron content has a negative feedback inhibition mechanism. In iron-sufficient cells, NCOA4 is degraded by the ubiquitin–proteasome system, increasing iron storage in the form of ferritin [27]. In addition, NCOA4 is regulated by hypoxia-inducible factor (HIF), and cells with increased activity of HIF1α or HIF2α will increase the expression level of NCOA4 [28]. Moderate ferritinophagy is essential in maintaining stable intracellular iron content. However, NCOA4 overexpression may excessively activate ferritinophagy and increase intracellular free iron, which might trigger ferroptosis [29].

### Amino acid metabolism

System Xc^−^ is a reverse transporter protein located in the cell membrane, consisting of a light chain subunit SLC7A11 and a heavy chain subunit SLC3A2 linked by a covalent disulfide bond, mediating the exchange of extracellular cystine and intracellular glutamate in a ratio of 1:1. The one that performs the amino acid transport function is SLC7A11, while SLC3A2 is responsible for enhancing protein stability [30]. Intracellular cystine can be converted into L-cysteine in a reducing environment, which can be utilized as a raw material for the synthesis of glutathione (GSH) [31]. Glutathione peroxidase 4 (GPX4), a member of the selenium-containing GPX family, is an essential peroxide-degrading enzyme in the body. The antioxidant process can catalyze the conversion of toxic peroxides to non-toxic hydroxyl compounds by using GSH as a reducing agent [15]. Therefore, GPX4 is an important factor in the negative regulation of ferroptosis.

### Lipid metabolism

Lipid peroxidation is usually the process by which free radicals seize electrons from lipids leading to the accumulation of lipid peroxyl radicals and hydroperoxides, which is an essential point in initiating ferroptosis. The process usually affects polyunsaturated fatty acids (PUFAs), especially arachidonic acid (AA) and adrenoic acid (AdA) [32]. Free AA or AdA is successively generated by the action of Acyl-CoA synthetase long-chain family member4 (ACSL4) and lysophosphatidylcholine acyltransferase 3 (LPCAT3) to form AA/AdA-phosphatidyl ethanolamine (PE), and the whole process can be summarized as the production of CoA derivatives of PUFA and their binding to phospholipids [33]. Subsequently, under the regulation of lipoxygenases (LOXs), PUFA-PE is peroxidized to PUFA-PE-OOH. The accumulation of lipid peroxides destroys cell membranes and other structures, which exacerbates the development of ferroptosis [34, 35].

### Other signals involved in the regulation of ferroptosis

Hypoxia-inducible factors (HIFs) are essential factors that regulate oxygen homeostasis at the level of gene transcription and are involved in the regulation of many iron metabolism-related proteins. Hydroxylation by prolyl hydroxylases (PHDs) can lead to the degradation of HIF-α, and iron ions can play an auxiliary role in this process. HIFs-PHDs are involved in the regulation of several iron metabolism-related proteins, and HIF-1 can activate the transcription of TFR by binding to hypoxia response elements [36]. In astrocytes, upregulation of HIF-1α and HIF-2α expression significantly increases DMT1 and FPN1 expression [37]. Grx3 and Grx4 in the glutaredoxin system can bind Fe–S proteins together with GSH to form a complex that functions in antioxidation and maintenance of iron homeostasis [38]. Depletion of GSH can increase free iron in the cytoplasm, which in turn is deployed to iron-dependent PHDs, promoting the expression of ATF4 in the cytoplasm and ultimately inducing the occurrence of ferroptosis. New insights propose that HIF-PHDs are oxygen sensors associated with neuronal survival and that iron chelators prevent heme-induced neuronal ferroptosis in an in vitro model of cerebral hemorrhage by targeting and regulating HIF-PHDs, an iron-dependent enzyme, rather than by inhibiting the Fenton response [39]. Coenzyme Q10 is a lipid-soluble antioxidant and its reduced form can resist free radicals that mediate the process of lipid peroxidation. Ferroptosis suppressor protein 1 (FSP1), tetrahydrobiopterin (BH4), and dihydroorotate dehydrogenase (DHODH) are involved in regulating the production of panthenol and perform an effect in the process of cellular antioxidation. The FSP1/CoQ10/NAD(P)H signaling pathway is an antioxidant pathway that parallels the GPX4 system and strongly synergizes with GPX4 to inhibit phospholipid peroxidation. FSP1 can decrease the accumulation of lipid peroxides by converting ubiquinone (CoQ) to the antioxidant dihydroubiquinone (CoQH2) [40]. BH4 is a critical enzyme in the synthesis of CoQ precursors. Normal expression of the BH4/dihydrofolate reductase (DHFR) pathway serves an essential function in promoting the production of CoQ [41]. New research suggests that DHODH, a flavin-dependent enzyme in mitochondria, can also promote CoQH2 production and thus mitigate lipid peroxidation damage in mitochondria [42].

GSH is a crucial antioxidant in the body, and the System Xc^−^-mediated uptake of cysteine is an important source of GSH synthesis. Both tumor suppressor p53 and nuclear factor erythroid 2-related factor (Nrf2) can be involved in regulating System Xc^−^ function, but their roles are distinct. p53 interferes with cystine uptake by inhibiting SLC7A11 expression, which in turn promotes cellular ferroptosis [43]. In contrast, Nrf2 can translocate to the nucleus after stimulation by oxidative stress to promote the expression of antioxidant molecules SLC7A11 and HO-1, and consequently inhibit cellular ferroptosis [44].

## Iron metabolism in different types of neuronal cells

The blood–brain barrier is a material exchange-limiting structure composed of capillary endothelial cells, basement membrane, and astrocytes, which makes it impossible for iron ions from peripheral blood to enter the brain directly, but requires a special transport process [45]. Iron transport involves two transmembrane processes, located on both sides of the vascular endothelium: the parietal membrane on the blood side and the basement membrane on the brain side. Iron in cerebrospinal fluid exists mainly in the form of transferrin-bound iron (TBI) and non-transferrin-bound iron (NTBI) [46]. Most of the iron flows into the cells by binding to TF and TFR1, which is highly expressed on the luminal side of the vascular endothelium. A small fraction enters via a NTBI pathway, probably involving the iron transporter in the parietal membrane [47]. Intracellular iron flows out of the basal plane via Fpn1 or other transporters. The iron being released outside the cell may bind to transferrin or complex with citric acid, ATP, etc., to form low molecular weight complexes, which are subsequently taken up by other cells [48]. Iron is capable of moving between different neurons and glial cells, but the exact mechanism is not yet fully clarified [49].

Different glial cells mainly express different iron regulatory proteins, such as astrocytes mainly express Fpn1, oligodendrocytes mainly express TFR1, and microglia mainly express ferritin [50]. Although all nerve cells can take up non-transferrin-bound iron, different nerve cells have different capacities to store iron. In an in vitro study comparing the ability of neurons, astrocytes, and microglia to accumulate iron, it was found that the ability of neuroglia (especially microglia) was stronger than that of neurons [51]. However, brain astrocytes in vivo express less ferritin and have a poor capacity to store iron [52]. As a major component of the blood–brain barrier, astrocytes serve a critical role in regulating iron homeostasis in the central nervous system. The presence of ferrous iron-converting enzymes on astrocytes can reduce Fe^3+^ to Fe^2+^, which is then transported into the cells via DMT1 [53]. Fpn1, hepcidin, and ceruloplasmin expressed in the terminal peduncle are all involved in regulating the transport of Fe^2+^ to the extracellular compartment in astrocytes [54, 55]. In addition, astrocytes appear to be more resistant to iron overload-induced neurotoxicity than other neuronal cells, which may be associated with the presence of their antioxidant system [52].

Microglia are neuroimmune cells with both anti-inflammatory and pro-inflammatory effects. The different effects depend mainly on the subpopulation into which they are activated: typically activated microglia (M1 type) and alternatively activated microglia (M2 type). M1 type is capable of producing pro-inflammatory cytokines such as IL-1, TNF-α, and IL-β, which in turn trigger cell damage [56]. Conversely, M2 secretes anti-inflammatory cytokines such as IL-10 and neurotrophic factors, which can exert anti-inflammatory and pro-neural tissue repair effects [57]. There are also differences in the regulation of iron transport in different states of microglia. The expression of DMT1 is upregulated in the M1 state, where it takes up more NTBI at the cell surface as a result of increased extracellular acidification [58]. In addition, the increase in extracellular acidification and the decrease in intracellular hemoglobin levels are changes that exacerbate the increase in intracellular labile iron. In the M2 state, the expression of TFR1 and ferritin increased, the cellular uptake of transferrin-bound iron increased, and the iron ions entering the cells could bind to ferritin to form stable complexes [59]. This avoids cell damage caused by elevated free iron outside the nerve cells. Iron metabolism in microglia is not deeply examined, and it is generally believed that iron uptake increases and output decrease after activation [60]. Iron in oligodendrocytes is mainly in the form of ferritin and transferrin [61]. Oligodendrocytes and choroid plexus are the main cells that synthesize transferrin, and iron in nerve cells is mainly delivered by transferrin. Therefore, the concentration of transferrin in cerebrospinal fluid can reflect the availability of iron in the brain [48]. In addition, oligodendrocytes can provide large amounts of iron for axonal myelin formation [62].

## Interaction of ferroptosis, neuroinflammation, and PND

Although the etiology of perioperative neurocognitive disorders remained unclear, neuroinflammation has been characterized as one of the major causes, especially in elderly patients. In addition, there is a complex link between ferroptosis and enzymes involved in inflammation, and ferroptosis inhibitors have demonstrated benefits in certain diseases due to their anti-inflammatory activity. Microglia perform an instrumental role in neuroinflammation as major mediators of the innate immune response, and their iron metabolism has been characterized as previously described. Iron overload is strongly associated with neuroinflammation. Elevated intracellular iron levels promote M1 polarization in microglia and facilitate a switch from M2 to M1 phenotype [63]. PUFAs and their metabolites are essential regulators of inflammation production and regression [64]. Arachidonic acid (AA) can produce leukotrienes (LT) via the lipoxygenases (LOXs) pathway and prostaglandins(PG) via the cyclooxygenases (COXs) pathway [65]. Ferroptosis appears to be linked to inflammation through regulation of the activity of LOXs and COXs. Lipid peroxidation is an important driver of ferroptotic cell death, and it has been identified in early studies that endogenous peroxisomal tension can regulate cellular lipoxygenase activity [66]. COX, as the rate limiting enzyme that catalyzes the conversion of AA to PG, has two isozymes, COX-1 and COX-2. Among them, COX-2 is an inducible enzyme encoded by PTGS2, which can activate inflammatory cells and thus trigger subsequent inflammatory responses. The emerging research has revealed that iron death can directly upregulate the expression of PTGS2 and thus promote the secretion of inflammatory mediators [67]. Elevated pro-inflammatory cytokines function in both the innate immune system and the adaptive immune system. Prostaglandin E promotes the differentiation of helper T cells and the secretion of TNF in dendritic cells, which in turn activates T cells [68]. Neuroinflammation is also capable of further exacerbating iron accumulation. Nuclear factor-kappa B (NF-κB) is a transcription factor that mediates the immune response and its activation contributes to iron accumulation in dopaminergic neurons [69]. Both neuroinflammation and ferroptosis exacerbate the disruption of neuronal cell function, which subsequently leads to cognitive dysfunction. Numerous studies have found that the application of iron chelators in mouse models of perioperative neurocognitive impairment suppresses neuroinflammation and enhances behavioral and cognitive performance.

## Association of ferroptosis with perioperative neurocognitive disorders

### Surgical trauma-induced changes in iron homeostasis of nerve cells

In research exploring the pathogenesis of POCD, there were significant differences in iron homeostasis in the hippocampus of splenectomized animals on the first and third postoperative days, but no significant changes were observed in the control and anesthesia-only groups. Iron accumulation was accompanied by increased oxidative stress, mainly in the form of increased malondialdehyde, a product of lipid peroxidation, and decreased levels of superoxide dismutase activity. The investigators proposed that postoperative cognitive decline might be principally attributed to surgical trauma rather than anesthesia and that changes in neuronal iron homeostasis and oxidative stress damage caused by surgical procedures were significant factors in cognitive decline [70]. Similarly, in another study using an abdominal surgery model to assess the effects of surgical trauma on hippocampal iron homeostasis, surgery resulted in altered levels of iron content and expression of proteins related to iron metabolism for up to 14 days, with increases in hippocampal iron content, ferritin, and DMT1, and a significant decrease in Fpn-1 levels, compared to controls and sham surgery [71]. In addition, surgical procedures inevitably lead to increased production of reactive oxygen species (ROS) in the body, and large amounts of ROS can result in a disruption of the intracellular oxidative and antioxidant balance [72]. The reaction between ROS and PUFA of the cell membrane promoted the production of lipid peroxides, which eventually caused ferroptosis in the cells. Reactive lipid species (RLS) can be regarded as a product of the non-enzymatic and enzymatic lipid peroxidation of PUFAs. Large intracellular accumulation of iron content and oxidative stress damage may promote ferroptosis and thus lead to decreased cellular function.

### The mechanism by which general anesthetics affect ferroptosis

#### General anesthetics affect iron metabolism

The neurotoxicity of general anesthetics has been a topical issue in the field of anesthesia. Repeated and prolonged exposure not only affects the neurodevelopment of infants and children, but also may accelerate the neurological degeneration of elderly patients [5, 73]. Elevated iron in the brain is associated with neuronal dysfunction. Current opinion suggests that general anesthetics may induce cognitive deficits by exacerbating iron overload in neuronal cells. This can be obtained by the detection of intracellular iron and iron metabolism-related protein levels. In a study using developing pups and aged rats, both ketamine and sevoflurane exposure was found to cause cytoplasmic and mitochondrial iron accumulation in hippocampal neurons. Reduced IRP2 and TfR1 expressions and increased ferritin expression further indicated that general anesthetics interfered with the balance of iron metabolism in neuronal cells [74]. Wang et al. discovered that sevoflurane significantly upregulated the expression of H-ferritin and, l-ferritin and iron export protein in the hippocampus and cortex of aged mice, but the expression of the input-related protein TfR1 was significantly decreased. In contrast to the previous findings, the expression level of IRP2 did not change significantly, and the investigators suggested that IRP2 may not be involved in sevoflurane-induced disorders of iron metabolism [75]. Fe–S protein is a component of the mitochondrial respiratory chain and plays a key role in biological activities as an important electron carrier. Anesthetics may affect mitochondrial function by causing disturbances in iron metabolism. Interestingly, maternal sevoflurane anesthetic exposure during pregnancy reduced brain iron levels and inhibited myelin production in offspring mice leading to cognitive impairment, while iron supplementation therapy improved impaired cognitive performance [76]. Therefore, the impact of sevoflurane on iron metabolism in mice of different ages may not be identical.

It is widely accepted that anesthetics indeed induce an imbalance of iron homeostasis in neuronal cells by affecting iron metabolism, but the exact molecular mechanism is not clear. One of the main mechanisms by which general anesthetics act may be through inhibition of excitatory synapses and enhancement of inhibitory synaptic transmission, specifically by affecting the function of ligand-gated ion channels, such as glutamate receptors/GABA receptors and glycine receptor ion channels [77]. N-Methyl-d-aspartic acid receptor (NMDAR) is a subtype of the ionotropic glutamate receptor, and it has been proposed that NMDAR may be the crucial link between anesthetics and cellular iron overload [74, 78]. Prolonged exposure of developing neurons to general anesthetics such as sevoflurane and ketamine may be responsible for a compensatory increase in NMDAR expression [79, 80]. Dexras1, also known as RASD1, is a Ras family small G protein, and activation of NMDAR can regulate iron transport in neurons through Dexras1 [81]. Dexras1 complexed with DMT1 not only promotes the transmembrane uptake of extracellular iron, but also increases the release of iron from lysosomes [82–84]. Wu et al. further confirm that NMDAR/RASD1/DMT1 signaling may be one of the molecular mechanisms by which general anesthetics disrupt the balance of iron metabolism in hippocampal neurons [74].

#### General anesthetics affect amino acid metabolism and lipid metabolism

It was found that isoflurane exposure did not alter the expression levels of genes related to iron metabolism and transport, suggesting that other alternative iron metabolic pathways were involved in inducing cellular ferroptosis [85]. The System Xc^−^/GSH/GPX4 regulatory axis is an important pathway that negatively regulates ferroptosis, and a decrease in glutathione-dependent antioxidant defense may be an essential trigger for ferroptosis [67, 86]. SLC7A11 is the predominant subunit in System Xc^−^ that functions as an amino acid transporter. Beclin1 is a protein that can exert a vital regulatory role in cellular autophagy, and new insights suggest that it is not only involved in autophagy regulation, but also interacts with different protein partners to regulate multiple cellular processes in a non-autophagy-dependent manner [87, 88]. AMPK-mediated phosphorylation of BECN1 promotes BECN1–SLC7A11 complex formation and thus blocks System Xc^−^ activity to induce cellular ferroptosis [89]. In an isoflurane-treated human neuroblastoma cell line, the expression level of phosphorylated Beclin1 protein was found to be significantly upregulated and mediated the inhibition of System Xc^−^ function by binding to SLC7A11 in a complex. Isoflurane resulted in GSH depletion in neuronal cells in a concentration-dependent manner, ultimately inducing the development of ferroptosis [85]. Similarly, in Liu et al.’s study, SLC7A11 and GSH levels in the hippocampus decreased significantly after 6 h of 1.5% isoflurane exposure, but GPX4 levels did not change significantly, suggesting that isoflurane exposure may induce ferroptosis by affecting System Xc^−^ and cystine uptake but not GPX4 [90]. However, it was controversial whether isoflurane affects ferroptosis by modulating GPX4 levels. Another study revealed a significant decrease in GPX 4 mRNA and protein expression levels in primary cortical neuronal cultures from mice treated with 2% isoflurane [91]. Lipid peroxidation is an influential part of ferroptosis, which usually refers to the process of oxidation of cell membrane by ROS and thus damage to cell structure and function. Malondialdehyde (MDA) and 4-hydroxynonenal (HNE) are common lipid peroxidation products. The brain is highly sensitive to lipid peroxidation due to its high oxygen consumption and the fact that nerve cell membranes are usually rich in polyunsaturated fatty acids [92]. A possible mechanism for omega-3 polyunsaturated fatty acids to improve postoperative cognitive dysfunction is that their components can replace arachidonic acid in cell membrane phospholipids to compete for cyclooxygenase and lipoxygenase and reduce RLS-induced cellular damage [93]. It is found that exposure of in vitro cultured cortical neurons to isoflurane significantly increases cellular ROS levels, impairs mitochondrial membrane potential, and ultimately induces cell death [91]. When the intracellular balance between oxidation and antioxidation is disturbed, more ROS reacting with lipids is an important factor leading to a decrease in cell viability.

### Impact of other relevant factors in the perioperative period

Ferroptosis is a cell death process that catalyzes lipid peroxidation of cell membranes in the presence of divalent iron or lipoxygenase, thereby destroying structure and function. Surgical trauma and anesthetic drugs may be the main factors contributing to postoperative cognitive decline. However, many other relevant factors in the perioperative period deserve equal attention. Decreased cerebral oxygen levels, inadequate cerebral perfusion due to prolonged hypotension, excessive blood loss, duration of surgery, clinical history of cerebrovascular disease, and postoperative pain are all risk factors for the development of POCD [94, 95]. These factors may differentially stimulate the release of cellular inflammatory factors and exacerbate oxidative stress damage. It is worth noting that cellular ferroptosis does not exist independently, but is interconnected with oxidative stress, energy stress, endoplasmic reticulum response, lysosomal and mitochondrial functions, etc. [15, 96–98]. Currently, studies exploring ferroptosis and POCD are still in their infancy, and more studies are expected to explore the effects of perioperative-related factors on iron metabolism in neuronal cells.

## Evidence for attenuation of developmental and degenerative neurotoxicity through modulation of ferroptosis

It is estimated that more than 300 million people worldwide will undergo surgical treatment each year. However, the stimulation of multiple factors such as surgery and anesthesia may inevitably lead to neurocognitive impairment in the body. The developing and aging brain appears to be more susceptible to damage in the perioperative period. Developmental neurotoxicity usually refers to abnormal changes in the structure and function of the nervous system caused by exposure of the brain to certain toxic substances during development, and there is an age-related susceptibility to such toxicity, ranging from 1 to 2 weeks after birth in rodents such as mice and from mid-conception to 3 years after birth in humans [99]. However, it has been proposed that the period of susceptibility to developmental neurotoxicity is determined by the cycle of division and differentiation in which neurons are present. Since neurons in the dentate gyrus and olfactory bulb regions have the capacity for continuous regeneration, some brain regions in adult life forms remain vulnerable to anesthetic-induced neurotoxicity [100]. In addition, the degenerative neurological changes associated with aging make the elderly more susceptible to multifactorial stimulation of cognitive decline in the perioperative period, which can also be considered degenerative neurotoxicity. New insights suggest that ferroptosis is involved in the pathology of postoperative cognitive dysfunction [101, 102]. The evidence from experimental studies to date is summarized in Table 1.

**Table 1:** Evidence for attenuation of perioperative neurocognitive disorders through modulation of ferroptosis

Treatment	Processing conditions	Experimental conditions	Animal	References	Year	Effects
DFO	Mice were intracerebroventricularly administrated with 0, 0, 0.1, 0.5, 2.5, and 5 μg of DFO 3 days prior to microinjection of LPS	A mouse model of LPS-induced cognitive impairment	Male C57BL/6 mice aged 10–12 weeks	[99]	2015	Reduction of brain iron, prevention of neuroinflammation, attenuation of oxidative stress, and apoptosis
DFO	Mice were treated daily for 6 days with 100 mg/kg DFO intraperitoneally (i.p.)	A model of laparotomy under general anesthesia and analgesia (4% chloral hydrate (10 ml/kg, i.p.,) plus 0.1% lidocaine)	C57BL/6J male mice (12–14 months)	[64]	2016	Restoration of iron homeostasis in the hippocampus, protection against neuroinflammation and oxidative stress in the hippocampus, and prevention of surgically induced BDNF dysfunction and memory impairment
DFO	DFO (100 mg/kg daily for up to 6 days)	Rats were anesthetized with 5% chloral hydrate and underwent exploratory laparotomy	Eighteen-month-old male Sprague–Dawley rats	[100]	2016	Inhibition of hippocampal iron accumulation and microglial activation, which in turn improves spatial memory capacity in aged rats
DFP; DMT1i	DFP (75 mg/kg, intraperitoneally, or DMT1i (50 mg/kg, orally, was administered to the animals 1 h before GA daily for three consecutive days	Ketamine (75 mg/kg) intraperitoneally or 3% sevoflurane 2 h inhalation daily for three consecutive days can induce neurotoxicity and cognitive impairments	Sprague–Dawley rat pups at postnatal day (PND) 6 and 15-month-old male C57BL/6 mice	[67]	2020	DFP protects mitochondrial function by chelating iron accumulated in hippocampal cytoplasm and mitochondria, and DMT1i inhibits ferroptosis by blocking iron uptake
Fer-1	Pre-treated at day in vitro 7 with 1 μM Fer-1	6 h of 2% isoflurane exposure	Primary cortical neuronal cultures were prepared from embryonic/gestational day 15 or 16 Swiss Webster mice	[85]	2018	Attenuation of isoflurane-induced RLS generation, cell death, and mitochondrial dysfunction
Fer-1; dimethyl fumarate (DMF)	15 mg/kg DMF was administered (i.p.) 16 h before anesthesia; 1 pmol of Fer-1 was injected into the striatum immediately before anesthesia	The anesthesia group received isoflurane (0.5%, 1.0% and 1.5%) in 100% oxygen for 2 h, 4 h and 6 h, respectively	1-week-old male C57/BL6 mice	[84]	2021	Inhibition of ferroptosis and protection of mitochondrial function
Fer-1; or deferoxamine mesylate (DFOM); si ACSL4	SH-SY5Y cells were treated with ferrostatin-1 (10 µM) or DFOM (20 µM)for 12 h	4.1% sev for different durations (0, 2, 4, 6, or 12 h)	Human neuroblastoma cell line (SH-SY5Y)	[96]	2021	Inhibition of sevoflurane-induced ferroptosis through activation of AMPK/mTOR signaling pathway
SiMIB2	25μL/mouse	2.5% sevoflurane with complete oxygen for 2 h	Aging male C57BL/6 mice (15 months old)	[95]	2021	Enhancement of GPX4 stability and decrease of its ubiquitination, which can reduce hippocampal ferroptosis
Low-iron forage	Iron content: 15 mg/kg (low iron)	2% Sev plus 40% oxygen within the anesthetic box for 6 h	Thirty male C57BL/6 12-month-old mice	[68]	2021	Reduction of iron levels, inhibition of β-secretase 1 (BACE1) expression, and Aβ accumulation

Iron accumulation can affect cognitive performance in mice by inducing multiple pathways including mitochondrial dysfunction, increased lethality of ROS, dendritic reduction, β-amyloid accumulation, and tau hyperphosphorylation [103]. In Wang et al.’s study, a low-iron diet appeared to improve sevoflurane-induced cognitive deficits [75]. In recent years, it has been widely investigated whether the regulation of ferroptosis by several treatments can prevent or improve POCD. The three main approaches of inhibiting ferroptosis include the following: iron chelation, prevention of lipid peroxidation, and scavenging of lipid peroxides [104]. Currently, iron chelators and lipophilic antioxidants are widely accepted methods to inhibit ferroptosis. Deferoxamine (DFO) is a high-affinity iron chelator that is capable of binding Fe^3+^ and exerts neuroprotective effects through suppression of LPS-induced increases in iron levels and neuroinflammation [105]. In Li et al.'s study, DFO pretreatment not only improved surgically induced changes in iron homeostasis, but also effectively reduced reactive microglia activation and inhibited oxidative stress and neuroinflammation. The mechanisms involved may include downregulation of gp91phox levels to inhibit oxidative stress signaling and suppression of p38 MAPK activation to reduce the release of inflammatory factors [71]. Pan et al. concluded similarly that preoperative administration of 100 mg/kg DFO improved iron accumulation in the hippocampus, inhibited microglial activation, and thus improved postoperative spatial memory in aged rats [106]. However, it is worth noting that the presence of the blood–brain barrier may not allow much DFO to actually enter the neuronal cells [107]. Deferiprone (DFP) is another metal chelator used to address iron overload and has the potential to treat a variety of diseases associated with iron accumulation. Its shuttle properties allow it to cross the blood–brain barrier and bind iron at multiple intracellular sites [108]. It was found that deferiprone pretreatment improved cognitive impairment induced by sevoflurane or ketamine, and behavioral testing ability was significantly improved in both adolescent and aged rats [74]. Cognitive impairment is strongly associated with iron overload and neuroinflammation, and DMT1 has been implicated as a key link between iron signaling and immunity [109]. DMT1-specific inhibitor (DMT1i) may attenuate GA-induced neurotoxicity by inhibiting iron uptake and counteracting compensatory upregulation of NMDAR [74]. Unlike the iron chelators mentioned above, ferrostatin-1 (Fer-1) is an inhibitor for ferroptosis with a lipophilic antioxidant effect. It could attenuate RLS generation, cell death, and mitochondrial dysfunction induced by isoflurane exposure [91]. Isoflurane can affect system Xc^−^ function and mitochondrial electron respiratory chain activity in a time-dependent and dose-dependent manner, and cysteine deprivation-induced hippocampal ferroptosis is an essential contributor to isoflurane-induced developmental neurotoxicity. Both ferroptosis inhibitor (ferrostatin-1) and mitochondria activator (dimethyl fumarate) modulate ferroptosis induced by isoflurane exposure, except that dimethyl fumarate (DMF) pretreatment decreases the activity of the complex IV in mitochondrial ETCs, but ferrostatin-1 does not have the effect [90]. Notably, the primary target of DMF is Nrf2, a key regulator of redox reactions. However, significant off-target effects of DMF have been found in studies of CNS diseases, which may limit the further application of DMF. The ability of iron chelators and lipophilic antioxidants to exert neuroprotective effects by inhibiting ferroptosis has also been demonstrated with human in vitro experiments. Furthermore, it was proposed that silencing of ACSL4 attenuated sevoflurane-induced ferroptosis by activating the AMPK/mTOR signaling pathway. Transfection of si-ACSL4 eliminated the inhibition of SH-SY5Y cell viability by sevoflurane [102]. In recent years, it was suggested that the E3 ubiquitin-protein ligase Mind bomb-2 (MIB2) could be involved in regulating neuronal function. In a sevoflurane-induced POCD model, knockdown of MIB2 enhanced the stability of GPX4 and reduced its ubiquitination, consequently reducing hippocampal ferroptosis [101]. The silencing of ACSL4 and MIB2 has enriched the approaches to inhibit ferroptosis in neuronal cells, and these findings provide new directions for the application of related drugs or strategies such as anti-ACSL4 and anti-MIB2 for the treatment of POCD.

## Conclusion and perspectives

Ferroptosis is a novel form of programmed cell death that is dependent on the accumulation of intracellular iron, resulting in inadequate antioxidant protection of the cellular lipid membrane. Ferroptosis is fundamentally different from apoptosis. The key to the apoptotic process is caspases, whose activation is achieved mainly through the death receptor pathway and the mitochondrial pathway. While a large body of literature in the past has emphasized apoptosis as a key cell death mechanism in the perioperative period after surgical or anesthetic exposure, emerging evidence supports the possible involvement of ferroptosis in mediating the pathogenesis of perioperative neurocognitive impairment. This implies that therapeutic measures related to the inhibition of ferroptosis may be promising agents for improving cognitive decline.

Iron is an essential trace element for the vital activity of human cells, however, the overload of iron ions can cause impairment in neuronal function. Experimental animal studies have found that ferroptosis is involved in the pathology of perioperative neurocognitive disorders. The current investigation showed that retreatment with the iron chelators deferiprone and deferoxamine together with the lipophilic antioxidant ferrostatin-1 all inhibited the ferroptosis process in neuronal cells and improved the cognitive behavior of experimental animals. Downregulation of ACSL4 and MIB2 can both independently inhibit sevoflurane-induced ferroptotic cell death. However, the presence of the blood–brain barrier may reduce the amount of iron chelator that actually enters the nervous system, and the binding of iron to iron chelator in cells at non-lesioned sites may also bring about many side effects. Discovering effective techniques for delivering biological agents to the central nervous system is a challenge in treating almost all neurological disorders. Studies to inhibit ferroptosis to improve perioperative neurocognitive impairment are still in their infancy, and the reproducibility and accuracy of experimental findings remain to be further validated. In addition, most of these current experiments have been conducted in mice. Provided that the ethics of animal experimentation are met, further experiments could be considered in primates that are more closely related to humans to obtain data more compatible with human clinical trials. Overall, there are relatively few studies in this area and more high-quality studies are warranted in the future to explore the possibility of inhibiting ferroptosis to improve cognitive impairment. Drugs that can treat cognitive dysfunction-related disorders by regulating ferroptosis deserve further development and exploration.

## Data Availability

All data generated or analyzed during this study are included in this article.
